# Diagnosis and treatment of hyperextension bicondylar tibial plateau fractures

**DOI:** 10.1186/s13018-019-1220-z

**Published:** 2019-06-25

**Authors:** Ruibo Zhao, Zhangyuan Lin, Haitao Long, Min Zeng, Liang Cheng, Yong Zhu

**Affiliations:** Department of Orthopedic Trauma, Xiangya Hospital, Central South University, No. 87 Xiangya Road, Changsha, 410008 Hunan China

**Keywords:** Tibial plateau fracture, Bicondylar, Hyperextention, Anterior midline incision

## Abstract

**Purpose:**

To report the diagnosis, injury mechanisms, and imaging characteristics of hyperextension bicondylar tibial plateau fractures and examine the indications and feasibility of the modified anterior midline incision as a treatment strategy.

**Methods:**

We performed a retrospective analysis of 11 cases of hyperextension bicondylar tibial plateau fractures who were treated with open reduction and internal fixation, predominantly via an anterolateral and posteromedialdouble incision or a modified anterior midline incision. Radiological and functional evaluations were performed.

**Results:**

Eleven patients were followed-up for a mean period of 11.5 months (range 3–24 months). The mean time to radiographic bony union was 12.5 weeks (range 10–26 weeks). At final follow-up, the average Rasmussen functional score was 26.8 (range 24 − 29); five patients had an excellent rating, and six a good rating. The average range of motion of the affected knees was 3.4–130° postoperatively. Fixation failure was not observed in any of the treated fractures.

**Conclusion:**

Hyperextension bicondylar tibial plateau fractures show a special Tiankeng-like collapse characteristic, while the changes in posterior tibial slope angle are easy to overlook. The modified anterior midline incision is a safe and effective approach for treatment of hyperextension bicondylar tibial plateau fractures with less rear displacement. Open reduction and double plating for the treatment of hyperextension bicondylar tibial plateau fractures provides excellent results.

## Background

Hyperextension bicondylar tibial plateau fractures are a particular form of tibial plateau fracture with a low incidence [1]. Although this is a Schatzker VI type tibial plateau fracture, it has distinct injury mechanisms, fracture morphology, and combined injuries. At present, there are only a few reports on this type of fracture, with a variety of terminologies including “over-extensile injured-type tibial plateau fractures,” “anterior tibial plateau fracture–dislocation,” and “hyperextension varus bicondylar tibial plateau fractures” [1–3]. In accordance with the injury mechanisms and radiological features, we suggest that this injury should be named “hyperextension bicondylar tibial plateau fractures.” We defined this injury as a compression fracture of the anterior tibial plateau caused by over-extension force of the knee joint, often accompanied by a tension fracture of the posterior tibial plateau, which is characterized by a reduced or reversed posterior tibial slope angle (pTSA).

The purpose of this study was to report the diagnosis, injury mechanisms, and imaging characteristics of hyperextension bicondylar tibial plateau fractures and to examine the indications and feasibility of the modified anterior midline incision as a treatment strategy.

## Materials and methods

### Patients

From April 2016 to February 2018, 12 cases of hyperextension bicondylar tibial plateau, fractures were treated in our Department of Orthopedics. The study was carried on with the approval of institution’s ethical review board. One patient suffered from severe articular cartilage destruction and was not included in our study as it was treated by total knee arthroplasty. There were six cases of fresh fractures, four cases of old fractures, and one case that was revised by osteotomy and internal fixation because of pain and instability of the knee. There were eight men and three women, with an age range of 19–61 years (average age, 42.4 years). The duration between injury and operation was 27.5 days (5–90 days). The duration between injury and operation of the six fresh fractures was 7.2 days (5–13 days). According to the Schatzker classification, all cases were Schatzker VI type fractures.

### Methods

All patients underwent X-ray and three -dimensional computed tomography examination of the knee joint before operation, to measure the displacement of the tibial plateau joint surface, the pTSA, and the medial tibial plateau angle (mTPA). Four patients received magnetic resonance imaging (MRI) examination, while all patients received deep intravenous doppler ultrasound examination. Patients with suspected popliteal artery injury underwent computed tomography angiography (CTA) examination of the lower extremities. All patients with fresh fractures received staged treatment. After admission, patients underwent bone traction or trans-articular fixation. Three patients with popliteal artery injury underwent saphenous vein grafting to repair the popliteal artery. All patients underwent open reduction and internal fixation if the soft tissue condition of the affected limb was suitable for surgery. All patients were operated by the same senior surgeons.

### Surgical technique

After nerve block anesthesia or genanral anesthesia was completed, each patient was placed in the supine position, with a balloon tourniquet on the proximal thigh and a round pillow under the popliteal fossa, which facilitated the opening of the fracture space in front of the tibial plateau fracture. Prophylactic antibiotics were given intravenously at 30 min before the incision. All fractures were fixed with anatomical locking compression plates (Depuy Synthes, USA).

The surgical approach was selected according to the degree of displacement of the posterior tibial cortex; fractures with no or minimal displacement of the posterior tibial cortex (usually ≤2 mm) were operated using the modified anterior midline incision, while fractures with a large displacement (usually > 2 mm) were operated by anterolateral and posteromedial double incisions. Only one case of hyperextension valgus bicondylar tibial plateau fracture with ipsilateral femoral shaft fracture was treated by a parapatellar anterolateral incision.

### Modified anterior midline incision (Figs. 1 and 2)

A longitudinal incision was performed parallel to the anterior midline of the knee, starting from the upper edge of the patellar, passing the lateral edge of the tibial tuberosity and approximately 1 cm lateral to the anterior tibial spine, and then extending approximately 7 cm distally. The full-thickness flap was protected, directly exposed to the deep fascia, stripped to both sides, and then exposed to the anterior edge of the fibular head laterally and the anterior edge of the superficial medial collateral ligament (MCL) medially. The flap was carefully retracted, and the lateral patellar retinaculum was incised, with a tibial tuberosity osteotomy performed occasionally to increase the anterior exposure, to obtain a 50–70-mm-long, 20-mm-wide proximal-end, 15-mm-wide distal end, and 10-mm-thick bone fragment in relation to its insertion in the tibial tubercle. The bone flap was then retracted proximally. Next, the anterior tibial muscle fascia was cut longitudinally, and the tibial is anterior muscle retracted laterally. A medial subperiosteal dissection was then used to reveal the medial fracture line. If the articular surface of the tibial plateau had a gap or step-off, the medial and lateral menisci were elevated to inspect the underlying joint surface. For restoration of sagittal plane alignment, disimpaction and elevation of the anterior metaphysis was performed by pulling the proximal metaphysis of the tibia forward and pulling the distal end of the tibia distally with the knee in the flexion position. The periosteal elevator was often used to reduce the collapsed fragments.

**Fig. 1:**
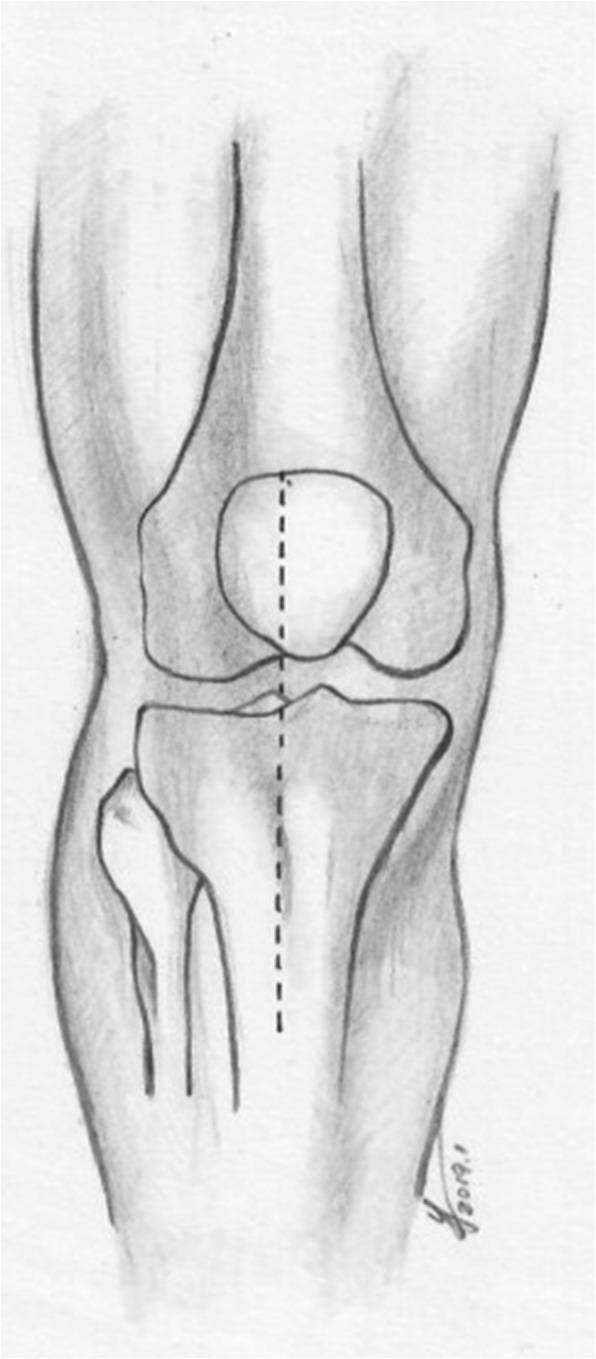
Modified anterior midline incision

**Fig. 2:**
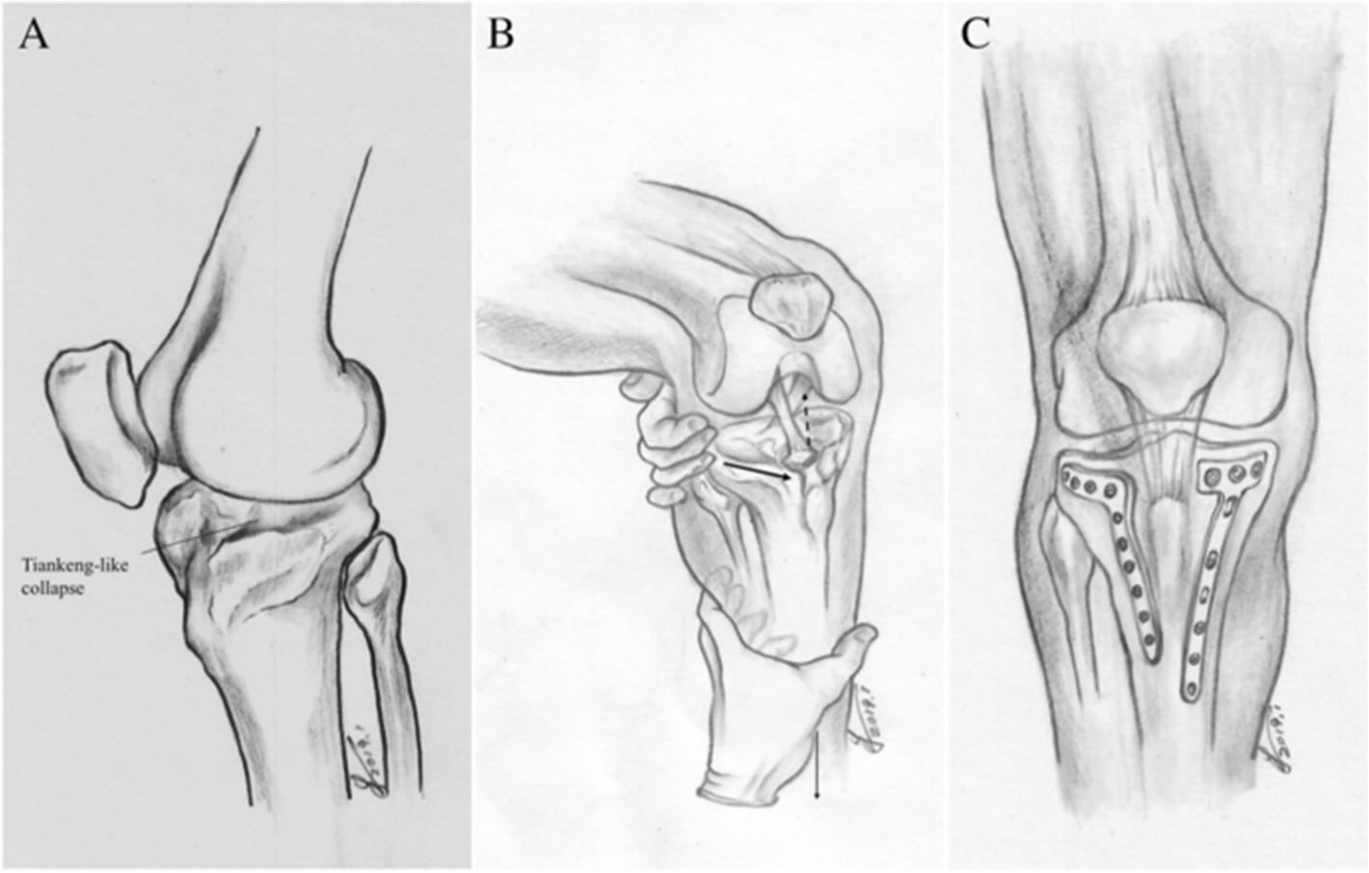
Schematic diagram describing fracture characteristics, reduction technique and fixation method. **a** Tiankeng-like collapse. **b** reduction technique. pulling the proximal metaphysis of the tibia forward(thick arrow) and pulling the distal end of the tibia distally(thin arrow), Tiankeng-like collapsed fragment would be reduced by ligamentotaxis(dotted arrow). **c** Double plates to fix hyperextension bicondylar tibial plateau fractures

After reduction was accomplished, the Kirschner wire was used for temporary fixation. Allograft bone was used for most cases. The C-arm was adopted to confirm the reduction, and a T-shaped locking plate was placed in front of the MCL medially, and an L-shaped locking plate was placed on the lateral side, to fix the fracture. Once again, the C-arm was used to confirm that the articular surface was smooth, the pTSA was recovered, and the position of internal fixation was satisfactory. The wound was flushed with saline, the drainage tube was indwelled, and the deep fascia was sutured intermittently. The parapatellar retinaculum and the anterior tibialis fascia were repaired, and the superficial fascia and skin was intermittently sutured in favor of drainage.

### Anterolateral and posteromedial double incision

Double incision surgery was performed mainly according to Firoozabadi’s method. After reduction was confirmed by X-ray, a T-shaped locking plate was then placed on the medial side of the tibial platform, and an L-shaped locking plate was placed on the lateral side.

### Rehabilitation and follow-up

Postoperatively, patients were instructed to begin range of motion movement of the knee on the first postoperative day. All patients were kept non-weight bearing for three months to avoid early loss of reduction. Toe-touch weight bearing was initiated at 12 weeks. Full weight bearing was not permitted until radiographic signs of primary bone healing, usually present at 12 weeks postoperatively.

All patients were followed for at least three months. The average follow-up was 11.5 months (3–24 months). Standard AP and lateral radiographs were taken to evaluate the mTPA, pTSA, and articular step-off. At the last follow-up, objective knee evaluation and patient-reported functional outcomes were assessed using the Rasmussen knee evaluation system. Complications, including infection, wound irritation, and neurovascular injury, were also noted.

### Statistical analysis

Statistical analysis was performed using SPSS, version 12.0(SPSS Inc, Chicago, IL, USA). Postoperative outcomes were retrieved and analyzed. Data are presented as mean ± standard deviation (SD). *P* values < 0.05 were considered to be statistically significant.

## Results

There were eight men and three women included in the study. Five fractures were treated by double incision, five fractures treated using the modified anterior midline incision, and one fracture with ipsilateral femoral shaft fracture treated using a lateral parapatellar incision. The patient’s demographics, injury mechanism, and fracture types are shown in Table 1.

**Table 1:** Demographic and peri-operative characteristic of our case series

No	Sex/age	Injury mechanism	Displacement of posterior cortex (mm)	Approach	Complication	Schatzker classification	Implant	Our classification
1	M/48	vechicle accident	3.6	Dual	MMR, LWI	VI	LCP	Hyperextention varus
2	M/36	Motor bike accident	2.4	Dual	LCL injury	VI	LCP	Hyperextention varus
3	F/34	Heavy crush	8.3	Dual	LCL injury, MMR, LMR, PAE	VI	LCP	Hyperextention varus
4	M/19	vechicle accident	19	AL	PAE	VI	LCP	Hyperextention varus
5	M/40	Falling from a car	0	MAM	–	VI	LCP	Pure hyperextention
6	M/61	Falling from height	2	MAM	DVT	VI	LCP	Pure hyperextention
7	M/39	vechicle accident	2	MAM	LMR, PAE, CPNI	VI	LCP	Hyperextention varus
8	F/61	Falling from height	0	MAM	DVT	VI	LCP	Hyperextention varus
9	F/26	vechicle accident	2	MAM	LMR	VI	LCP	Pure hyperextention
10	M/51	Motor bike accident	0	PL	–	VI	LCP	Hyperextention valgus
11	M/51	vechicle accident	2.2	Dual	–	VI	LCP	Pure hyperextention

*M* male, *F* female, *MAM* modified anterior midline incision, *AL* anterolateral incision, *MMR* medial meniscus rupture *LWI* late wound infection, *LMR* lateral meniscus rupture. *PAE* popliteal artery embolism, *DVT* deep vein thrombosis, *CPNI* common peroneal nerve injury, *LCP* locking compression plate

Of the 11 patients, three were accompanied by popliteal artery rupture or embolism, three lateral menisci ruptures were observed at surgery, and one lateral menisci was diagnosed by MRI. Other concomitant injuries are shown in Table 1.

Radiological findings at immediately postoperatively and at final follow-up were assessed using the Rasmussen anatomical scoring system. There were no significant differences in mTPA, pTSA, or articular step-off on the radiographs between immediately postoperatively and at final follow-up (*P* < 0.05). At final follow-up, the average range of motion of the affected knees was 3.4–130°. Further, according to the Rasmussen functional score, the average functional score was 26.8(24–29), with five patients ranked excellent, and the remaining six ranked as good. The results of measurements and analyses are shown in Tables 2 and 3. The mean time to radiographic bony union was 12.5 weeks (range 10–26 weeks).

**Table 2:** peri-operative and final follow-up measurements of our case series

No.	Pre-op		Post-op					Final follow-up						
	mTPA	pTSA	Depression	Condylar widening	Angulation	mTPA	pTSA	Depression	Condylar widening	Angulation	mTPA	pTSA	ROM	RS
1	83.32	− 19.01	0	0	1.92	88.92	9.46	0	0	1.94	88.94	9.02	9 to 133	26
2	83.52	− 10.16	2	0	− 0.85	86.15	5.27	2	0	− 0.85	86.10	5.20	0 to 131	29
3	81.02	− 15.59	0	5	0.27	87.27	2.80	0	5	0.27	87.27	2.80	0 to 130	26
4	78.93	− 34.41	2	0	− 4.75	82.25	1.15	2.1	0	− 4.75	82.25	1.15	19 to 112	24
5	88.44	− 10.64	0	5	− 3.76	83.24	13.33	0	5	− 3.76	83.34	13.30	− 2 to 135	28
6	87.02	− 7.64	0	4.7	4.92	91.92	− 1.27	0	4.9	4.92	91.76	− 1.24	2 to 113	29
7	81.44	− 27.42	4	7	− 7.23	79.77	− 9.87	4	7.4	− 7.23	79.79	− 9.88	− 2 to 145	29
8	83.52	− 10.15	1	4	1.27	88.27	6.93	1	4	1.27	88.20	6.87	5 to 145	28
9	89.55	− 16.74	0	8	5.38	92.38	4.28	0	9	5.38	92.35	4.06	4.2 to 122	26
10	90.02	− 0.07	0	0	− 3.1	83.90	5.94	0	0	− 3.1	83.80	5.76	0 to 132	24
11	87.57	− 25.06	2.8	0	4.47	91.47	− 3.52	2.8	0	4.47	90.47	− 3.47	2.6 to 132	26

*mTPA* medial tibial plateau angle, *pTSA* posterior tibial slope angle, *ROM* range of motion, *RS* Rasmussen score

**Table 3:** Statistical analysis of measurements of our case series between postoperative and final follow-up

	Post-op	Final follow-up	Stasticas
mTPA	86.87 ± 4.21	86.75 ± 4.08	*t* = 1.271 *P* = 0.233
pTSA	3.14 ± 6.39	3.05 ± 6.32	*t* = 1.941 *P* = 0.081
Depression	1.07 ± 1.42	1.08 ± 1.43	*t* = − 1 *P* = 0.341
Condylar widening	3.06 ± 3.13	3.21 ± 3.35	*t* = − 1.551 *P* = 0.152
Angulation	− 0.13 ± 4.21	− 0.13 ± 4.22	*t* = − 1 *P* = 0.341

**Fig. 3:**
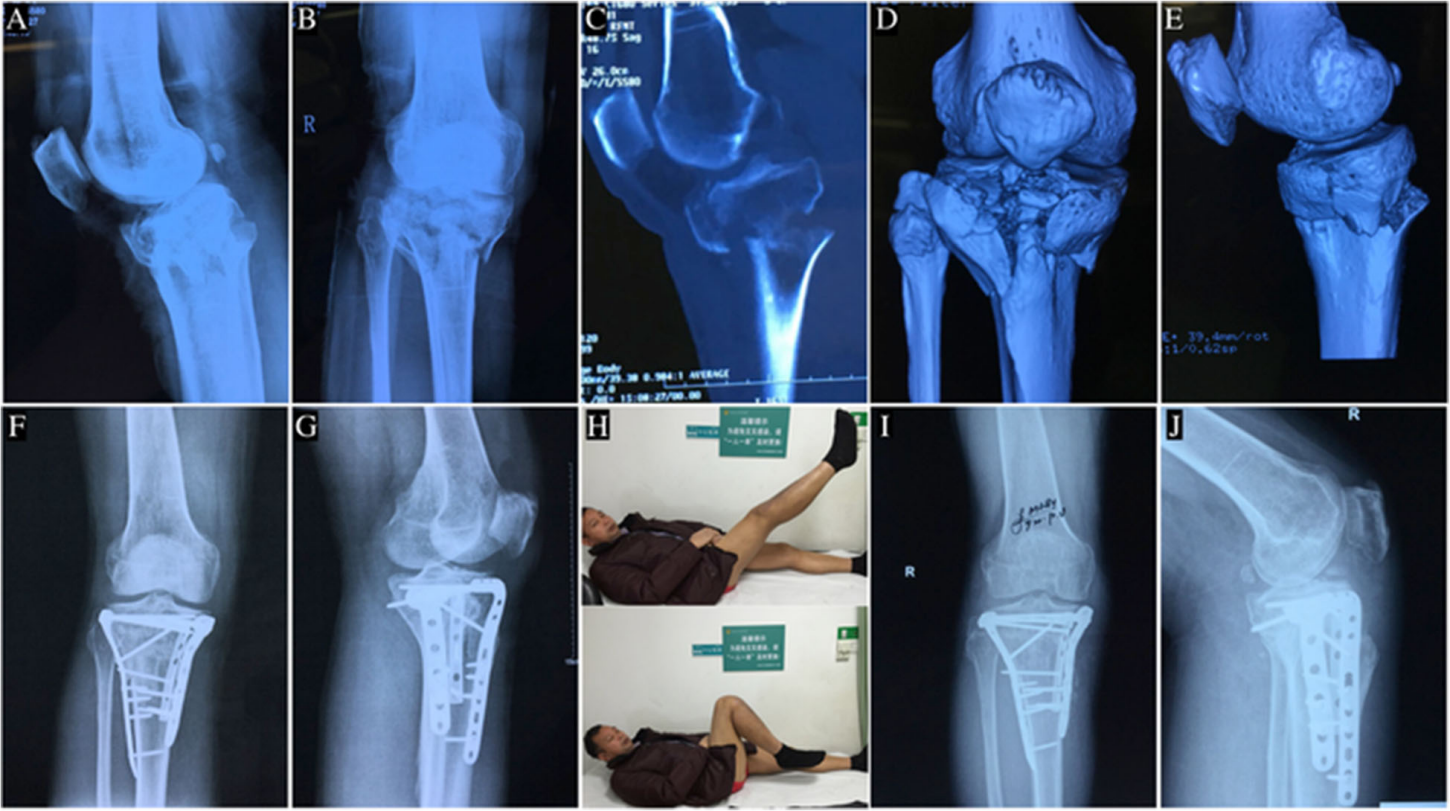
A 48-year-old man with hyperextension bicondylar tibial plateau fracture treated with anterolateral and posteromedial double incisions. **a** Preoperative anteroposterior view of X-ray. **b** Preoperative lateral view of X-ray shows a reversed pTSA. **c**,**d**,**e** Three-dimensional reconstruction CT shows collapse of the anterior part of tibial plateau and a large displacement of the posterior tibial cortex. **f**,**g** Postoperative anteroposterior view and lateral view of X-ray. **h** The function of the knee has recovered at the 9-month follow up. **i**,**j** Anteroposterior view and lateral view of X-ray show the fracture has healed at the 9-month follow up

**Fig. 4:**
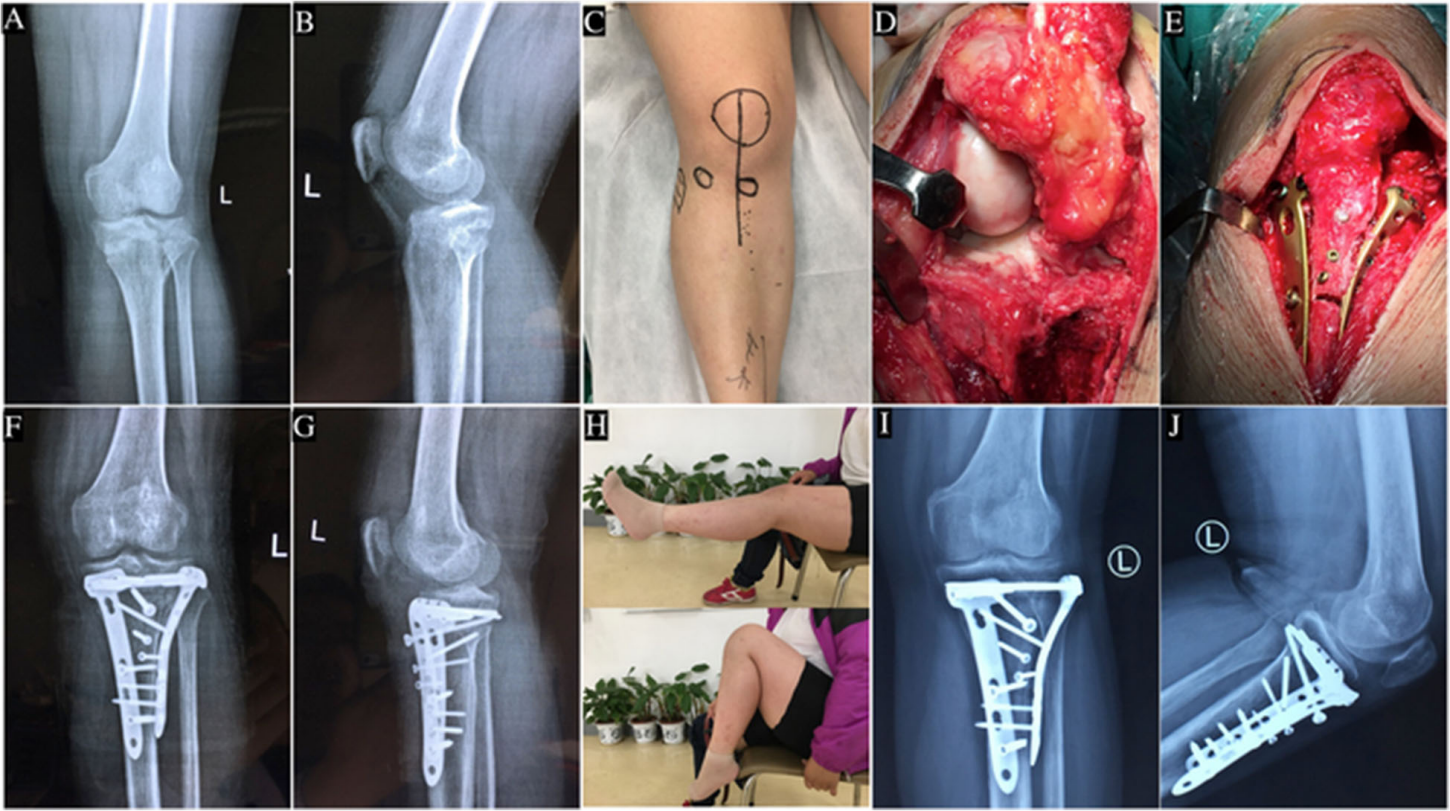
A 26-year-old woman with an old hyperextension bicondylar tibial plateau fracture treated with modified anterior midline incision and tibial tuberosity osteotomy. **a**,**b** Preoperative anteroposterior view and lateral view of X-ray. **c** Modified anterior midline incision. **d** The whole Tiankeng-like collapsed fragment can be exposed after the osteotomized tibial tuberosity is retracted proximally. **e** Intraoperative photograph shows the fracture was fixed with double plates and osteotomized tibial tuberosity was fixed with 3cortical screws. **f**,**g** Postoperative anteroposterior view and lateral view of X-ray. **h** Range of motion of the knee is close to normal at the 3.5-month follow up postoperatively. **i**,**j** Anteroposterior view and lateral view of X-ray show no reduction loss occurred and the fracture has healed 3.5 months after surgery

One patient had a subcutaneous hematoma at four days after the operation. The wound healed without skin edge necrosis and infection after dressing and drainage for several days. One dual-incision patient developed a delayed type of infection at nine months of recovery. Antibiotic treatment failed to control the infection, which was finally cured by plate removal. Two patients developed deep vein thrombosis (DVT) and received placement of a temporary filter. The typical cases were shown in Figs. 3 and 4.

## Discussion

Hyperextension bicondylar tibial plateau fractures are a particular form of tibial plateau fracture with a low incidence [1]. So far, there are few reports on this type of fracture, with a variety of terminologies used. In 2007, Chen et al. [2] proposed the concept of over-extensile injured-type tibial plateau fractures, which are believed to be caused by over-extension and squeezing of the femoral condyle to the anterior side of the tibial plateau, resulting in a collapse of the anterior side of the tibial plateau and a change in the posterior tilt angle of the tibial plateau. However, in that study, the frontal fragment of the tibial plateau was very thin, resulting in a poor grip of the screw on bone fragments. Further, even if a bone graft is added to enhance fixation, it is difficult to maintain a good reduction, so it was suggested that the majority of these fractures do not require surgery.

In recent years, this type of injury has become of increasing interest. In 2016, Wu et al. [1] reported 18 such cases (termed anterior tibial plateau fracture–dislocation), which was considered to be a special Schatzker VI type tibial plateau fracture. These fractures were characterized by anterior subsidence of the tibial component and irreducible dislocation of the knee joint and were often associated with neurovascular and ligament injury around the knee, and a high incidence of complications during fixation surgery. Good results can be achieved by open reduction and internal fixation (ORIF) via an anterolateral and posteromedialdouble incision. In 2016, Firoozabadi et al. [3] also reported 25 cases of hyperextension varus bicondylar tibial plateau fractures, a fracture pattern with an anterior compression fracture through the metaphysis of the proximal tibia, creating a sagittal plane deformity in which the normal posterior slope of the tibial articular surface is decreased or reversed, tension failure of the posterior cortex, and various deformities on the coronal plane. In that study, the incidence of popliteal vessel injury and common peroneal nerve injury was as high as 12% and 16%, respectively, and 12% of patients had unilateral compartment syndrome. All patients were treated by ORIF with double incision, and all had bone grafting.

In Luo’s adapted three-column theory, Wang et al. suggested that combined medial and lateral column fractures are usually caused by axial violence with the knee in extension. Implant placement at both the anterolateral and anteromedial sides is necessary to maintain the pTSA. Further, the position of the buttress plate should be determined in accordance with the mTPA [4]. The terminology used for this pattern of injury is not currently uniform. However, based on its mechanism of injury and radiological features, we suggest naming the fracture as hyperextension bicondylar tibial plateau fracture.

### Classification and associated injury

In accordance with the change in mTPA, we divided hyperextension bicondylar tibial plateau fractures into hyperextension varus type (mTPA < 85°), pure hyperextension type (85° ≤ mTPA ≤ 90°) and hyperextension valgus type (mTPA > 90°). It would have been preferable to classify these types by comparing the mTPA of the injured side with that of the contralateral side. However, X-ray of the normal knee was not performed in some patients. Thus, we compared the mTPA of the injured side with normal population data (85–90°) [5]. The hyperextension varus type is highly combined with popliteal artery injury, common peroneal nerve injury, and lateral collateral ligament injury. In the present study, of the six cases of hyperextension varus-type fractures of the tibial plateau, three were complicated with popliteal artery embolism, which is higher than the reported incidence of 12% and 22.2% [1, 3]. There were only two cases with lateral collateral ligament injury in this group, although the actual injury rate may be higher as we did not routinely perform MRI on all patients. The reported incidence of common peroneal nerve injury is as high as 16% [3], while we only observed one case with such injury out of our 11 patients. Meniscus injury occurred in four of 11 cases, three of which were diagnosed during open surgery and were repaired at that time.

These classifications can also be used to instruct the main plating. In principle, the main plate of the hyperextension varus type of tibial plateau fracture should be placed on the anterior medial side, while for the hyperextension valgus type the main plate should be placed on the anterolateral side, and for the pure hyperextension type, both sides should be fixed equally.

### Surgical indications

Surgical indications for bicondylar tibial plateau fractures vary in the literature [1, 2, 6]. Most authors believe that surgery should be performed if the intra-articular step-off or gap exceeds 2 mm [2, 6]. It was also suggested that a pTSA change > 5°from a normal reference value of 5° should be considered “clinically significant” [7]. By contrast, an angular deformity of > 10° in the sagittal plane was reported as a surgical indication [6]. Further, an angular deformity of > 10° in the coronal plane, a metaphyseal-diaphyseal translation of > 1 cm, open fracture, associated compartment syndrome, associated ligament injury requiring repair, and an associated fracture of the ipsilateral tibia or fibular are also surgical indications of bicondylar tibial plateau fractures [6]. Based on our present findings, the displacement can even fail to meet these criteria. Nevertheless, if the knee is certified to be unstable by the lateral stress test, the procedure should be performed. Our series was mainly based on Hall’s indication criteria [6].

### Approach

In previous reports, anterolateral and posteromedial double incisions are typically used for hyperextension bicondylar tibial plateau fractures [1, 3]. For fractures with a large displacement of the posterior tibial cortex that require reduction under direct visualization, the posteromedial incision provides excellent exposure of the posteromedial fragments, and facilitates placing of the posterior supporting plate. However, a disadvantage of this approach is that it requires massive stripping of soft tissue to expose the anterior compression fracture, and if the anterior buttressing plate is placed, it is inconvenient to insert screws. Thus, for hyperextension type tibial plateau fractures with no or little posterior displacement, we developed a modified anterior midline incision. The traditional anterior midline incision provides excellent exposure of the whole anterior part of the tibial plateau, although it is associated with more sensory disturbances of the anterior aspect of the knee than that for anterolateral incisions [8]. The traditional anterior midline incision, which usually passes through the medial edge of the tibial tuberosity, is not in line with the anterolateral incision of the knee. However, the single anterolateral approach is difficult to manage for medial plateau fractures. Thus, we combined the advantages of both incisions in our modified anterior midline incision approach.

As most of the hyperextension-type bicondylar tibial plateau fractures are characterized by central depression of the anterior part of the medial plateau, lateral plateau, and the median eminence as a whole, while the cortices of the plateau may lie in situ (similar to the geographic fracture “Tiankeng”), we termed this central depression as “Tiankeng-like collapse.” It is inconvenient to observe the Tiankeng-like collapse from the side, but easy to expose the whole Tiankeng-like collapse from the window behind the patellar ligament. Thus, the patellar ligament can be retracted medially or laterally to reveal the Tiankeng-like collapse through the modified anterior midline incision. For severely comminuted cases or old fractures, tibial tuberosity osteotomy can be performed to increase the anterior exposure of the modified anterior midline incision.

Although there is evidence of a high incidence of poor wound healing for treatment of bicondylar tibial plateau fractures with an anterior median incision, this may be associated with the unhealthy soft tissue caused by the high energy injury and massive stripping of soft tissue required to expose the posterior parts of both the medial and lateral tibial plateau [9]. None of our five cases had wound infection or skin necrosis. This low incidence of infection may be associated with our strict control of indications, gentle operation to protect the skin flaps, and limited peeling range. We also performed full drainage and moderate pressure dressing, which may reduce the occurrence of subcutaneous hematoma in front of the knee joint.

### Fixation

Because of anterior depression of both the medial and lateral plateau, we typically use double plates to fix hyperextension bicondylar tibial plateau fractures. However, most of these fractures are characterized by a Tiankeng-like collapse, as the medial and lateral platforms and the median eminence are collapsed as a whole. Thus, if the depression fragment is not comminuted, it may be fixed by a single plate, although this approach should be certificated by mechanical testing and clinical follow-up. In our series, one case of hyperextension valgus-type fracture was treated with a single lateral locking plate, with no loss of reduction observed at recent follow-up.

### Limitations

Because of the low incidence of hyperextension bicondylar tibial plateau fractures, the sample size of our study was small. Further, during the case analysis, the revision cases, old fracture cases, and fresh fracture cases were analyzed together, which may have affected our findings. Finally, the follow-up time was short, and further long-term assessment of our cases is required.

## Conclusion

Hyperextension bicondylar tibial plateau fractures show a special Tiankeng-like collapse characteristic, while the changes in pTSA are easy to overlook. The reduction should be performed by ligamentotaxis reversal of the injury mechanism, so as to correct the pTSA and mTPA. The appropriate surgical approach is selected in accordance with the degree of displacement of the posterior tibial cortex. The modified anterior midline incision is a safe and effective approach for treatment of hyperextension bicondylar tibial plateau fractures with less rear displacement. Open reduction and double plating for the treatment of hyperextension bicondylar tibial plateau fractures provide excellent results.

## Data Availability

Data sharing is not applicable to this article as no datasets were generated or analyzed during the current study.

## Abbreviations

CTA: Computed tomography angiography
DVT: Deep vein thrombosis
MCL: Medial collateral ligament
MRI: Magnetic resonance imaging
mTPA: Medial tibial plateau angle
ORIF: Open reduction and internal fixation
pTSA: Posterior tibial slope angle
